# Cinnamic Acid and Caffeic Acid Effects on Gastric Tight Junction Proteins Analyzed in *Xenopus laevis* Oocytes

**DOI:** 10.3390/membranes14020040

**Published:** 2024-02-01

**Authors:** Marie-Luise Vollstädt, Laura Stein, Nora Brunner, Salah Amasheh

**Affiliations:** School of Veterinary Medicine, Institute of Veterinary Physiology, Freie Universität Berlin, 14163 Berlin, Germany; marie-luise.vollstaedt@fu-berlin.de (M.-L.V.); laura.stein@fu-berlin.de (L.S.);

**Keywords:** claudins, epithelial barrier, *Xenopus* oocytes, tight junction, cinnamic acid, caffeic acid, heterologous expression, stomach

## Abstract

Analysis of secondary plant compounds for the development of novel therapies is a common focus of experimental biomedicine. Currently, multiple health-supporting properties of plant-derived molecules are known but still information on many mechanisms is scarce. Cinnamic acid and caffeic acid are two of the most abundant polyphenols in human dietary fruits and vegetables. In this study, we investigated cinnamic acid and caffeic acid effects on the gastric barrier, which is primarily provided by members of the transmembrane tight junction protein family of claudins. The *Xenopus laevis* oocyte has been established, in recent years, as a heterologous expression system for analysis of transmembrane tight junction protein interactions, by performing paired oocyte experiments to identify an effect on protein–protein interactions, *in vitro*. In our current study, human gastric claudin-4, -5, and -18.2. were expressed and detected in the oocyte plasma membrane by freeze fracture electron microscopy and immunoblotting. Oocytes were paired and incubated with 100 µM or 200 µM cinnamic acid or caffeic acid, or Ringer’s solution, respectively. Caffeic acid showed no effect on the contact area strength of paired oocytes but led to an increased contact area size. In contrast, cinnamic acid-incubated paired oocytes revealed a reduced contact area and a strengthening effect on the contact area was identified. These results may indicate that caffeic acid and cinnamic acid both show an effect on gastric barrier integrity via direct effects on tight junction proteins.

## 1. Introduction

The vertebrate gastric mucosa consists of a single layer of epithelial cells. Neighboring cells are interconnected by tight junction (TJ) strands [1] and form the intrinsic epithelial barrier. This barrier depends on the sealing properties of the TJs. The critical components of TJ strands are a family of integral membrane proteins, namely claudins, with 27 members and splice variants currently known, which are expressed in a tissue-specific way [2,3,4]. Claudins are the essential proteins regarding regulation of permeability, barrier function, and polarity of epithelial layers [5,6,7]. They are tetraspan proteins with two extracellular loops [8], and the interaction of these loops between the adjacent epithelial cells is called *trans*-interaction [9]. In healthy gastric mucosa, claudin-18.2 (cldn 18.2) [10], claudin-4 (cldn 4) [11], and claudin-5 (cldn 5) [12] are primarily responsible for determining the epithelial barrier. Many exogenous factors trigger mucosal damage and epithelial erosion. These include dietary nitrates, food allergens and contaminants, metals, chemicals, radiation, and pharmaceuticals like nonsteroidal anti-inflammatory drugs. Moreover, commonly diagnosed *Helicobacter pylori* infections induce chronic gastritis and can provoke gastric cancer, both leading to TJ dysfunction and decreased barrier properties [13,14].

A variety of secondary plant compounds indicate a capability for the treatment of chronic diseases, including gastrointestinal inflammation [15]. One common polyphenol, cinnamic acid, occurs naturally in several human dietary components like cinnamon, grapes, whole grains, spinach, celery, and honey [16,17]. The structure of cinnamic acid, with an aromatic core, conjugated double bond, and hydroxyl group, manifests its antioxidant function but cinnamic acid provides numerous biological qualities including antimicrobial [18,19,20], anticancer [21], neuroprotective [22], anti-inflammatory, and antidiabetic properties [23]. The same characteristics apply to hydroxycinnamic acids like caffeic acid [24,25]. It is similarly a common compound in a variety of dietary plants like apples, plums, lingonberries, black chokeberries, and herbs of the mint family, e.g., sage, thyme, oregano, and spearmint [26].

However, information about the effects of dietary secondary plant compounds on epithelial barrier properties is rather limited. The aim of the present study was to investigate the direct effects of cinnamic acid and caffeic acid on stomach-specific claudin–claudin *trans*-interaction.

Therefore, we employed an established two-cell model, using African claw frog, *Xenopus leavis* oocytes as a heterologous expression system [27], for analyses of the human gastric claudins cldn 4, cldn 5 and cldn 18.2 *trans*-TJ strand interaction.

## 2. Materials and Methods

### 2.1. Animals

Care and treatments of *Xenopus laevis* were permitted by the animal welfare officer of Freie Universität Berlin and accredited by the State Office for Health and Social Affairs (Landesamt für Gesundheit und Soziales Berlin, Germany, permit E 0061/23) in accordance with German legislation guidelines. 

### 2.2. Chemicals

Cinnamic acid (Sigma Aldrich, Taufkirchen, Germany) and caffeic acid (Sigma Aldrich, Taufkirchen, Germany) were dissolved for preparing 40 mM stock solutions, which were stored at −20 °C. Directly before clustering the oocytes the stocks were attenuated with ORi to 100 μM and 200 μM. 

### 2.3. Oocyte Harvest and cRNA Microinjection

**Figure 1:**
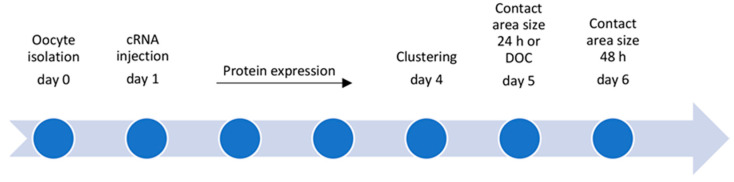
Flow chart: experimental procedures from oocyte isolation to functional analyses (day 0–6, DOC: double orbital challenge).

For isolating the oocytes, adult female *Xenopus laevis* were anesthetized with a 0.2% MS222 (ethyl 3-aminobenzoate methanesulfonate, Sigma Aldrich, Taufkirchen, Germany) bath solution for 10 min at 20 °C. Once the rightening reflex was gone, ovarian tissue was taken by surgical laparotomy. Therefore, the skin and muscle in the lower lateral abdomen were cut, the ovarian lobes were surgically removed and placed in oocyte Ringer solution (ORi). For removing the connective tissue and follicular cell layers, the exteriorized ovarian mass was treated with 1.5 mg/mL collagenase (Fisher BioReagents BP2649-1, Fisher Scientific, Schwerte, Germany) dissolved in ORi for 90 min at room temperature as described by Vitzthum et al. [27]. Remaining follicular cells were eliminated by incubation in Ca^2+^-free ORi, pH 7.4 for 10 min on a shaker with 50 rpm. Oocytes in stages of development V and VI (<1000 µm in diameter) with a regular round shape and steady pigment coating were collected. Dissolved in an injection volume of 50.6 nl, 1 ng cRNA encoding human cldn 4, cldn 5, and cldn 18.2 was injected per oocyte (Nanoliter 2010, World Precision Instruments, Sarasota, FL, USA). For the next 3 days, the injected oocytes were stored in ORi at 16 °C for protein expression before clustering and analysis (Figure 1). 

### 2.4. Immunoblotting of Membrane Fractions

Three days after injection, 10 injected oocytes were collected and lysed in a homogenization buffer (MgCl_2_ (5 mM), NaH_2_PO_4_ (5 mM), EDTA (ethylenediaminetetraacetic acid) (1 mM), sucrose (80 mM), and Tris (Tris(hydroxymethyl) aminomethane) (20 mM; pH 7.4). After homogenizing, the suspension was centrifugated two times at 200 rpm for 10 min at 4 °C. Next, the supernatant was centrifugated again at 13,000 rpm for 30 min at 4 °C, resulting in a pellet containing the membrane fraction. Pellets were resuspended in 80 μL homogenization buffer for colorimetric protein quantification by using Pierce 600 nm Protein Assay Kit (Thermo Fischer Scientific, Henningsdorf, Germany). Membrane proteins were quantified in a 96-well plate with bovine serum albumin standards (Thermo Fischer Scientific, Henningsdorf, Germany) from 125 to 2000 μg/mL by a plate reader (PerkinElmer EnSpire Multimode Plate Reader, Waltham, MA, USA) adjusted to 562 nm. Samples were prepared for immunoblotting by adding 4 × Laemmli buffer (Bio-Rad Laboratories, Munich, Germany), and urea (9 mol/L, Carl Roth GmbH, Karlsruhe, Germany) and denaturing for 8 min at 50 °C. They were loaded onto a 10% SDS polyacrylamide gel and electrophoresed. PVDF membranes blocked with 5% non-fat dry milk in Tris-buffered saline for 120 min were used for protein transfer. Cldn 4, cldn 5 and cldn 18.2 were detected with specific primary antibodies (Invitrogen #32-9400, #35-2500, #34-1600, #700178, Life Technologies, Carlsbad, CA, USA) overnight at 7 °C. The following day peroxidase-conjugated goat anti-rabbit and anti-mouse antibodies (#7074, #7076 Cell Signaling Technology, Danvers, MA, USA) were used for binding the primary antibodies by incubation for 45 min at room temperature. After adding detection solution (Clarity Western ECL Blotting Substrate, #1705061, Bio-Rad Laboratories GmbH, Munich, Germany) for visualization of protein signals, a ChemiDoc MP system (Bio-Rad Laboratories, Munich, Germany) was used.

### 2.5. Freeze Fracture Electron Microscopy

Freeze fracture electron microscopy was conducted as reported by Greene et al. [28]. Overnight injected oocytes were fixed by incubating in glutaraldehyde (2.5% in 0.1 M cacodylate buffer) at 4 °C. After washing with cacodylate buffer, samples were incubated in 30% glycerol and frozen in liquid nitrogen for cryoprotection. Platinum and carbon (BAF400D; Balzers, Liechtenstein) were used for fracturing and shadowing. By washing in sodium hypochlorite remaining organic material was removed. With transmission electron microscopy (EM-10, Zeiss, Oberkochen, Germany), oocytes were scanned and photographed with a digital camera (Tröndle GmbH, Singen, Germany). The TJ strands were morphometrically analyzed at a magnification of 20,000×.

### 2.6. Paired Oocyte Assay with Cinnamic Acid and Caffeic Acid Treatment

Three days after microinjection, 5–10 oocytes were transferred to a Petri dish containing ORi and Mannitol for shrinking the cells, to remove the vitelline membrane with two sharp forceps manually without damaging the plasma membrane. Devitellinized oocytes were transferred to a 24-well plate. One well contained 2 mL ORi with 100 or 200 micromol cinnamic acid or caffeic acid. Cldn 4-, cldn 5-, and cldn 18.2-expressing oocytes in ORi served as controls. Two oocytes were transported in one well, where they were pushed together carefully. For 48 h, paired oocytes were kept in well plates at 16 °C. At 24 h and 48 h after pairing, the contact area diameter was measured by bright-field microscopy (DMI6000 B Microscope, LAS AF software 3.0 Leica Microsystems, Wetzlar, Germany) using the micron scale. As the contact areas are circular, they were calculated using the circle equation $A=\pi r^{2}$.

### 2.7. Double Orbital Challenge

The vitelline membranes were removed as described in 2.5. Two oocytes expressing cldn 4, cldn 5, and cldn 18.2 were paired in one well of 24-well plates filled with 1 mL ORi with 100 μM or 200 μM caffeic acid or cinnamic acid, respectively. Claudin-injected paired oocytes in ORi served as controls. After storing at 16 °C for 24 h, a double orbital challenge was performed to examine the contact strength. First, the length of the contact area was measured by transmitted light microscopy. Subsequently, a 24-well plate with two paired oocytes per well was placed in a Perkin Elmer Enspire bracket for a double orbital shaking treatment (120 s, 200 rpm, diameter 1 mm). After double orbital challenge, the contact length was measured again and the change in the contact area was calculated.

### 2.8. Statistical Analyses

Statistical Analysis was performed with JMP Pro 16.0.0 (SAS Institute Inc., Cary, NC, USA). Paired oocyte assay data are depicted as means and standard error of the mean (SEM), the controls are defined as 100%. Data from the double orbital challenge are presented as box plots, representing the first quantile (25%), the median (50%), and the second quantile (75%). In addition, the whiskers are displayed for the 10th and 90th percentile. n is the number of oocyte pairs. For checking normal distribution, the Shapiro–Wilk test was used. Kruskal–Wallis test was carried out for non-parametric data followed by a Dunn–Bonferroni correction. Values of *p* < 0.05 are determined as statistically significant (presented as * *p* < 0.05, ** *p* < 0.01.

## 3. Results

### 3.1. Detection of Heterologously Expressed Gastric Claudins in Xenopus Oocytes

**Figure 2:**
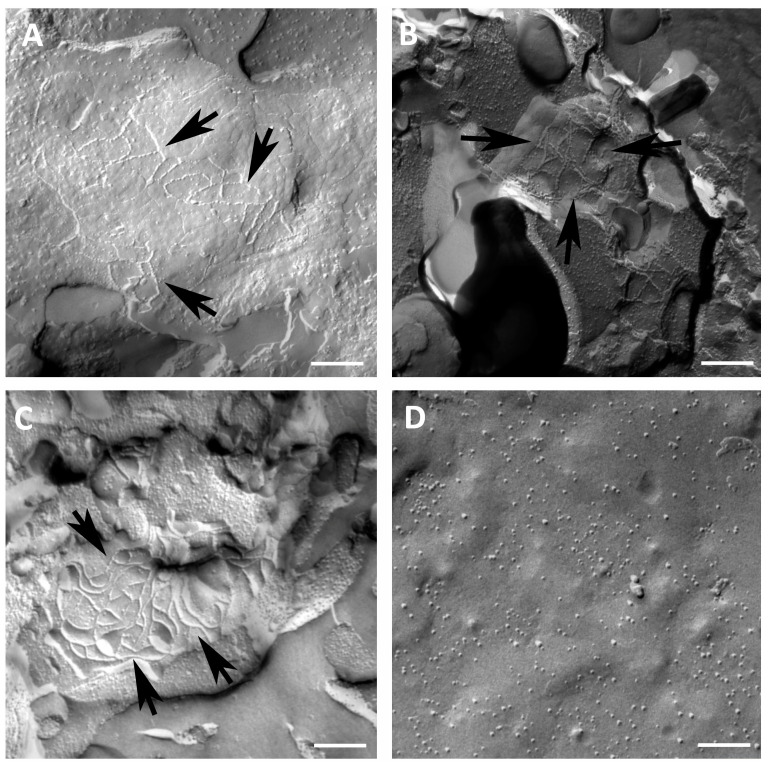
Freeze fracture electron microscopy showing formation of TJ strands in oocytes expressing cldn 4, cldn 5, or cldn 18.2, respectively (arrows). (**A**) Freeze fracture electron microscopy reveals TJ protein cldn 4 as an intermittent network of connected square strands; (**B**) freeze fracture electron microscopy reveals TJ protein cldn 5 as angular fibrils in *Xenopus laevis* oocytes; (**C**) freeze fracture electron microscopy of oocytes expressing cldn 18.2 reveals a meshwork of undulating strands with intersections; (**D**) water-injected control oocytes with a smooth surface, representative images of oocytes derived from three animals. Scale bar: 250 nm.

Freeze fracture electron microscopy revealed strand-forming structures in the plasma membrane of oocytes expressing cldn 4, cldn 5, or cldn 18.2, most notably in the protoplasmatic (P-) face of the membrane (Figure 2). Cldn 4-injected oocytes exhibited a network of connected, square strands (Figure 2A). In plasma membranes of oocytes expressing cldn 5, highly organized strands of an angular shape were displayed (Figure 2B). Likewise, cldn 18.2-injected oocytes showed undulating organized strands with a huge amount of intersections (Figure 2C), whereas control oocytes (injected with RNAse-free water) offered a smooth surface without detectable TJ strands (Figure 2D). 

### 3.2. Immunoblotting of Heterologously Co-Expressed cldn 4, cldn 5, and cldn 18.2 in Xenopus Oocytes

**Figure 3:**
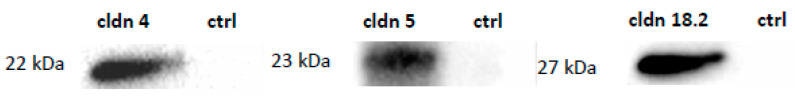
Immunoblots of heterologous co-expression of CLDN4/5/18. Claudins were detected to locate protein expression within the oocyte membrane. In contrast, no endogenous signals for the tight junction proteins were detected in controls (ctrl, representative images).

To verify the heterologous co-expression of human cldn 4, cldn 5, and cldn 18.2 in *Xenopus laevis* oocyte membranes, 3 days after injection of cRNA, immunoblotting was performed. The membrane fractions from three different animals exhibited specific protein signals at 22 kDa (cldn 4), 23 kDa (cldn 5), and 28 kDa (cldn 18.2), whereas oocytes injected with RNAase-free water (ctrl) showed no signals for these proteins. Hence, the heterologous co-expression of three different human stomach-specific claudins within the oocyte membrane was successfully detected (Figure 3). 

### 3.3. Paired Oocyte Assay Shows Cinnamic Acid and Caffeic Acid Effects on the Contact Area Size

**Figure 4:**
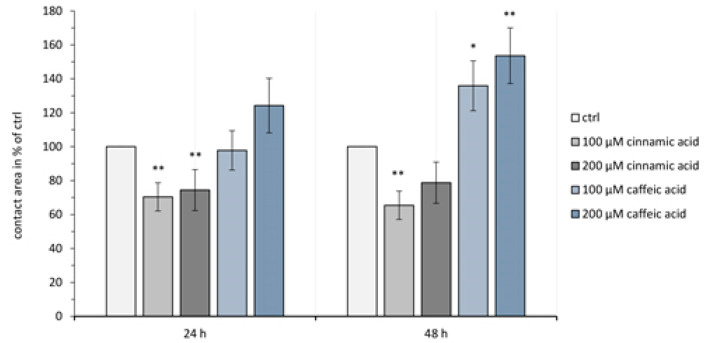
Contact areas of paired oocytes expressing cldn 4, cldn 5, and cldn 18.2. After 24 h there is no significant caffeic acid effect on the contact area size but after 48 h there is an increase in the contact area. In contrast, cinnamic acid caused a decrease in the contact area after 24 h. Data presented in means ± SEM (n = 13–21, * *p* < 0.05, ** *p* < 0.01.

The size of the contact area was affected by cinnamic acid and caffeic acid (Figure 4). At 24 h after pairing, the contact area size of the paired oocytes incubated in 100 µM cinnamic acid decreased to 70.43 ± 8.31 % (*p* = 0.0015, n = 21) compared to the control group (set to 100 %). Similarly, the contact area size was reduced in 200 µM cinnamic acid to 74.41 ± 12.06 % (*p* = 0.0089, n = 14). This effect was validated after 48 h. The contact area in 100 µM cinnamic acid decreased to 65.46 ± 8.36 % (*p* = 0.0013, n = 21) and in 200 µM cinnamic acid to 79.76 ± 12.14 % (*p* = 0.0527, n = 14) compared to controls set to 100 %. 

In contrast, caffeic acid led to an increased contact area. Here, 24 h after clustering, the contact area of oocyte pairs treated with 100 µM caffeic acid was 97.89 ± 11.58 % (*p* = 0.9421, n = 13), and after 48 h the contact area increased to 135.9 ± 14.71 % (*p* = 0.0347, n = 13). Equally, the higher concentration of 200 µM caffeic acid resulted in a contact area size of 124.21 ± 16.07 % (*p* = 0.3440, n = 15) after 24 h and 153.61 ± 16.41 % (*p* = 0.0093, n = 15) after 48 h. 

### 3.4. Double Orbital Challenge Revealed Cinnamic Acid-Dependent Increase in Contact Area Strength

**Figure 5:**
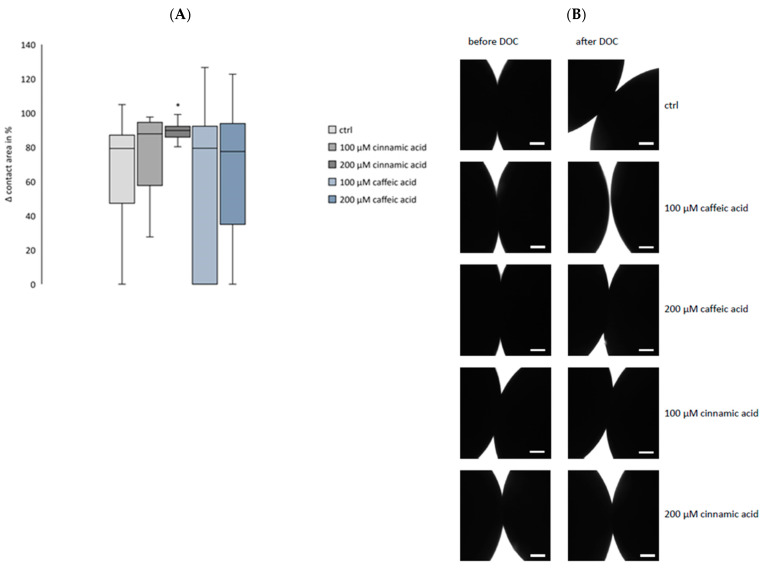
Change of contact area size before and after DOC (**A**) and visualization and quantification by transmitted light microscopy (**B**). Oocytes co-expressing cldn 4, cldn 5, and cldn 18.2 treated with 200 µM cinnamic acid retained a significantly higher contact area size after DOC compared to the ctrl. Similarly, 100 µM cinnamic acid led to a larger contact area but the difference compared to the ctrl was not significant. Both concentrations of caffeic acid did not affect the contact area size after DOC compared to the ctrl. (n = 11–16, Kruskal–Wallis test followed by Dunn–Bonferroni correction, * *p* < 0.05, representative images, scale bars = 200 µm).

At 24 h after pairing cldn 4, cldn 5, and cldn 18.2-expressing oocytes, a double orbital challenge (DOC) was performed to identify the cinnamic acid or caffeic acid effects on the contact area strength (Figure 5). The size of the contact area of the controls decreased to 65.97 ± 7.93 % (n = 16) after the DOC compared to their contact area size before the DOC. Cinnamic acid reduced this decrease. The oocytes treated with 100 µM cinnamic acid showed a decreased contact area size of 74.01 ± 7.98 % (*p* = 0.2873, n = 14), and 200 µM cinnamic acid led to a decrease of just 89.14 ± 1.81 % (*p* = 0.0421, n = 13). There was no caffeic acid effect regarding the contact strength. Clustered oocytes incubated in 100 µM caffeic acid showed a decreased contact area size of 64.43 ± 13.32 % (*p* = 1.0, n = 11), and in 200 µM caffeic acid, a decrease to 68.19 ± 11.37 % (*p* = 1.0, n = 13).

## 4. Discussion

The gastric epithelium, composed of a monolayer of epithelial cells, expresses sealing tight junction proteins, providing the structural correlate of barrier function. Claudins play a central role in this barrier and thus are essential for maintaining homeostasis in living organisms. Modeling of organ- and tissue-specific tight junction complexes by heterologous expression of claudins was shown in previous studies by our group, demonstrating the suitability of the oocytes for tight junction research [29,30]. The integral membrane components of the tight junction, such as claudins, interact with cytoplasmatic proteins of the zonula occludens (ZO) family linked to the actin cytoskeleton [31]. This implies that the presence of ZO proteins is crucial for the correct assembly of TJs [32]. More recently, endogenous expression of α+ and α− isoforms of ZO-1 were detected in *Xenopus laevis* oocytes at development stages V and VI [33]. Thus, the *Xenopus* oocyte is a convenient heterologous expression system for TJ proteins and enables a two-cell assay for the analysis of the protein–protein interaction or the claudin *trans*-interaction, between two neighboring cells. Freeze fracture electron microscopy revealed the ability of strand formation by every single claudin within the oocyte membrane and the immunoblot results showed a successful heterologous co-expression of cldn 4, cldn 5, and cldn 18.2. Consequently, the injected oocytes represent a model of gastric TJ strands. Most epithelial cells express various claudins; therefore, the examination of single or selected specific claudins in epithelial tissue is challenging. The presented *Xenopus laevis* oocyte model enables us to analyze and characterize stomach-specific claudins and their interactions isolated from tissue-dependent factors, making it possible to investigate the effects of secondary plant compounds directly on the *trans*-interaction of adjacent claudins.

Our current study presents a paired oocyte assay for the detection of cinnamic acid’s or caffeic acid’s effects on the integrity of tight junctions. 100 μM and 200 μM cinnamic acid led to a decreased contact area 24 h after clustering, and caffeic acid induced an increased contact area after 48 h. Moreover, the contact area strength was affected, further demonstrating cinnamic acid’s and caffeic acid’s effects on the *trans*-interaction of gastric claudins. To accomplish this, the double orbital challenge was performed for challenging the interaction within the contact area of the clustered *Xenopus* oocytes. At 24 h after pairing of cldn 4, cldn 5, and cldn 18.2-expressing oocytes, the DOC revealed the strengthening effect of 200 μM cinnamic acid on the contact area. This identified effect on the contact area by cinnamic acid indicates an even stronger effect on tight junction integrity. In contrast, there was no detected caffeic acid effect on the contact area strength. Apart from *trans*-interaction of claudins, *cis*-interaction and oligomerization of claudins occurs within the same membrane [34]; therefore, caffeic acid possibly increased the contact area size by affecting the *cis*-interaction of the heterologous co-expressed claudins in one cell. The enhancement of our oocyte model with heterologous expression of tagged claudins would enable us to analyze the claudin–claudin interaction directly and could be part of further studies.

The integrity of epithelial barriers does not only depend on the interaction between claudins but also on the interactions between claudins and scaffolding proteins like ZO-1, which directly binds actin and thus forms a stabilizing link between the seal and the cytoskeleton [35,36]. These bindings are not static but are described as highly dynamic [37]. Potentially, the detected effects on the contact area size and contact area strength of *Xenopus* oocytes expressing cldn 4, cldn 5, and cldn 18.2 by cinnamic acid and caffeic acid might include different effects of these compounds on the TJ-associated cytoskeleton. Regarding these effects, different compounds were previously shown to mediate effects on actin and TJ structure, e.g., incubation with *C. difficile* toxins TcdA and TcdB results in the disorganization of apical and basal F-actin and decreased association of ZO-1 with the actin cytoskeleton in an intestinal epithelial cell line [38]. Furthermore, an increased translocation of ZO-1 from TJ was observed within 2-3 h of toxin exposure [39]. In our study, the incubation with cinnamic acid and caffeic acid lasted 24 h in the DOC or 48 h in the paired oocyte assay; therefore, a compound-mediated effect on the TJ-associated actin cytoskeleton might not be excluded, especially because the endogenous expression of ZO-1 in unfertilized *Xenopus laevis* oocytes heterologously expressing claudins is already represented [33]. 

Thus, as one possible limitation of our study, the oocytes are solely employed as a heterologous expression system with a “saturated” translational machinery inducing a strong heterologous expression of the three tight junction proteins. To accomplish this, a maximum quantity of cRNA was injected to provide a stable surface expression of tight junction proteins for analysis of the direct effects on tight junction protein interaction. Therefore, an effect of regulatory mechanisms regarding expression level was not intended nor expected within the scope of this study, as well as any effects on the water-injected oocytes. Moreover, regulations provided through endogenous mechanisms, such as the cytoskeleton were not the focus of the current study. Although this is in accordance with our previous studies [29,30], these points might be addressed in subsequent analyses.

Another point would be that the freeze fracture electron microscopy images of oocytes expressing claudin 4, claudin 5, or claudin 18 served as a proof of concept that every single claudin is able to form tight junction strands. Specific detection of heterologously expressed combinations of claudins by freeze fracture electron microscopy might be also interesting for future approaches. However, for the time being, a combined expression was demonstrated by Western blotting and was also analyzed by immunofluorescence confocal microscopy [29].

Information about the effects of dietary secondary plant compounds on epithelial barrier properties is rather limited. Nevertheless, studies performed regarding secondary plant compound effects on barrier properties are promising. The isoflavones Biochanin A and prunetin improved the barrier tightness of intestinal epithelial CaCo-2/TC-7 cells [40], quercetin and its metabolite DHBA were shown to increase the epithelial resistance of Caco-2-cells [41], and the phenolic compound resveratrol restored the barrier integrity of human colonic HT-29/B6 cell monolayers infected with C. jejuni [42]. Moreover, Brassica oleracea sprouts’ juices, including several anthocyanins, quercetin-3-Glc, cryptochlorogenic, neochlorogenic, and cinnamic acids, were demonstrated to protect intestinal barrier integrity in Caco-2 cells exposed to tumor necrosis factor α [43]. Chen et al. [44] described an improved intestinal barrier function using a dietary supplementation of caffeic acid in weaned piglets by promoting tight junction protein-related gene expression, and our present study demonstrates that secondary plant compounds may also have direct barrier-strengthening effects on the gastric epithelium. However, further studies are required to fully reveal the potential of secondary plant compound effects on epithelial barrier properties and their perspectives to prevent or medicate barrier disorders in the gastrointestinal tract. 

## Data Availability

Data are contained within the article. More detailed datasets analyzed during the current study are available from the corresponding author upon reasonable request.
